# Association between muscle hydration measures acquired using bioelectrical impedance spectroscopy and magnetic resonance imaging in healthy and hemodialysis population

**DOI:** 10.14814/phy2.12219

**Published:** 2015-01-27

**Authors:** Anuradha Sawant, Andrew A. House, Bert M. Chesworth, Denise M. Connelly, Robert Lindsay, Joe Gati, Robert Bartha, Tom J. Overend

**Affiliations:** Western University, London, Ontario, Canada; London Health Sciences Center, University Hospital Campus, London, Ontario, Canada; Division of Nephrology, London Health Sciences Centre, Western University, London, Ontario, Canada; Department of Epidemiology and Biostatistics, School of Physical Therapy, Western University, London, Ontario, Canada; School of Physical Therapy, Western University, London, Ontario, Canada; The Centre for Functional and Metabolic Mapping, Robarts Research Institute, Western University, London, Ontario, Canada

**Keywords:** Apparent diffusion coefficient, end‐stage renal disease, hemodialysis, skeletal muscle, Transverse relaxation times

## Abstract

Establishing the effect of fluctuating extracellular fluid (ECF) volume on muscle strength in people with end‐stage renal disease (ESRD) on hemodialysis (HD) is essential, as inadequate hydration of the skeletal muscles impacts its strength and endurance. Bioelectrical impedance spectroscopy (BIS) has been a widely used method for estimating ECF volume of a limb or calf segment. Magnetic resonance imaging (MRI)‐acquired transverse relaxation times (*T*_2_) has also been used for estimating ECF volumes of individual skeletal muscles. The purpose of this study was to determine the association between *T*_2_ (gold standard) of tibialis anterior (TA), medial (MG), and lateral gastrocnemius (LG), and soleus muscles and calf BIS ECF, in healthy and in people with ESRD/HD. Calf BIS and MRI measures were collected on two occasions before and after HD session in people with ESRD/HD and on a single occasion for the healthy participants. Linear regression analysis was used to establish the association between these measures. Thirty‐two healthy and 22 participants on HD were recruited. The association between *T*_2_ of TA, LG, MG, and soleus muscles and ratio of calf BIS‐acquired ECF and intracellular fluids (ICF) were: TA:* β *= 0.30, *P* > 0.05; LG:* β *= 0.37, *P* = 0.035; MG:* β *= 0.43, *P* = 0.014; soleus: *β *= 0.60, *P* < 0.001. For the HD group, calf ECF was significantly associated with *T*_2_ of TA (*β *= 0.44, *P* = 0.042), and medial gastrocnemius (*β *= 0.47, *P* = 0.027) following HD only. Hence BIS‐acquired measures cannot be used to measure ECF volumes of a single muscle in the ESRD/HD population; however, BIS could be utilized to estimate ratio of ECF: ICF in healthy population for the LG, MG, and soleus muscles.

## Abstract (precis)

This figure summarizes the process of estimating the transverse relaxation time constants of a region of interest of tibialis anterior muscle before and after hemodialysis of a participant (computed using OsiriX 5.8.2 for Mac). Three small regions of interest were selected with care to avoid visible fatty tissue or muscle septum. The values of the three values thus generated were then averaged to estimate the representative transverse relation time constant for that muscle.

## Introduction

People with end‐stage renal disease (ESRD) on hemodialysis (HD) are known to have skeletal muscle weakness (Jamal et al. 2006) and renal osteo‐dystrophy (Sakhaee and Gonzalez 1999), predisposing them to increased risk for falls (Cook et al. 2006) and long bone fractures (Jamal et al. 2006). Falls commonly predict morbidity, mortality, and perhaps need for institutional care (Sattin 1992) in community dwelling older adults. Hence, exercise training has been strongly recommended to maintain muscle function and prevent falls and related injuries (Ikizler and Himmelfarb 2006). However, the presumed benefits of exercise in this population have not been observed consistently in interventional studies (Sawant et al. 2014).

Expansion of extra cellular fluid (ECF) volume is one of the manifestations of ESRD, and HD is required to correct this, although it may lead to periods of relative volume contraction (Plum et al. 2001). Clinical symptoms such as hypotension and intradialytic cramps have been associated with volume depletion or dehydration (Passauer et al. 1998), however, such HD‐related dehydration or volume depletion has not yet been linked to poor response to exercise interventions or muscle weakness in this population. Jain and Lindsay (2008) suggested that the majority of fluid loss and refilling takes place in the extremities to maintain central blood volume. Hence change in pre‐ and post‐HD whole body mass cannot be used to establish hydration of a limb segment or single muscle. The impact of this fluctuating hydration, pre‐and post‐HD, of the skeletal muscle in people receiving HD is yet to be elucidated. Since eccentric exercises or activities such as standing and walking in a volume contracted individual without kidney disease may exacerbate skeletal muscle damage, leading to impairment of structural and contractile properties (Cleary et al. 2006), it is important to assess the HD‐related changes in ECF volumes of skeletal muscle. Such investigations may provide insight and understanding of the effect of such fluctuating hydration on muscle function in people with ESRD/HD.

Total body water turnover is complex, hence, none of the measures of hydration based on laboratory techniques such as isotope dilution, neutron activation analysis, body mass change, and plasma volume change, have been established as “gold standard” measures to unquestionably represent accurate changes in total body water gain or loss (Armstrong 2007). Bioelectrical impedance spectroscopy (BIS), a valid and reliable method, has been widely used to measure ECF and intracellular fluid (ICF) space within a limb segment such as calf, limb or whole body (van Marken Lichtenbelt 1994). Researchers have utilized this method in people with ESRD/HD to evaluate the fluctuations in ECF and ICF before and after HD interventions (Kushner et al. 1996). However, whether BIS measures can be used to estimate hydration of a single muscle is yet to be established.

Magnetic resonance imaging (MRI)‐acquired transverse relaxation times (*T*_2_ – a measure of transverse magnetization signal decay) or apparent diffusion coefficients (ADC) have been used as an estimate of changing interstitial fluids/ECF volumes of skeletal muscle. Our earlier investigations in healthy populations have established that the reliability of *T*_2_ is excellent (ICC_2,1_: 0.9) whereas the same for ADC is poor to moderate (ICC_2,1:_ 0.6) (Sawant et al. 2013). Hence we chose to investigate the association between calf BIS (cBIS) measures and *T*_2_ for the purposes of this study. Hatakenaka et al. (2006) have demonstrated a relationship (Pearson r = 0.813, *P* < 0.05) between the ratio of extracellular and intracellular space and *T*_2_ in an animal model (rats), using biopsy to microscopically measure extra and intracellular spaces for establishing this correlation. Hence *T*_2_ can be considered as a valid estimate of the ratio of extracellular and intracellular space within a skeletal muscle; that is, *T*_2_ values will rise with an increase in extracellular space. Investigations using *T*_2_ for estimating changes in ECF volume following neural damage (Polak et al. 1988; Holl et al. 2008), and exercise (Nygren and Kaijser 2002) lend further support to use this measure as an estimate of ECF at the muscle/tissue level. However, *T*_2_ has not been commonly used as estimates of ECF in research (Yanagisawa et al. 2009). Acquisition and analysis of MRI‐based methods are both expensive and resource intensive.

The primary objective of this paper was to evaluate association between calf ECF measured using BIS (cBIS) and MRI‐acquired *T*_2_ of TA to establish if cBIS ECF can be used to estimate interstitial fluid in TA muscle instead of resource and labor intensive MRI‐acquired *T*_2_ in healthy and ESRD/HD population. Secondary objectives were to evaluate association between cBIS ECF and *T*_2_ of medial and lateral gastrocnemius, and soleus muscles to determine if cBIS ECF can be used to estimate interstitial fluid of these specific muscles in healthy and ESRD/HD population.

A priori hypotheses were grounded in theoretical assumptions based on distribution of type II fibers and the size/volume of the muscles. Previous reports have established presence of atrophy of type II fibers in people with ESRD/HD (Sawant et al. 2011). However, according to Johansen et al. (2003) the total muscle compartment for the TA was not significantly different in size from the control participants (people with no disease). As a result, the space occupied by these atrophic muscle fibers may expand the relative ECF volume of the muscle. Hence we hypothesized that *T*_2_ of the TA muscle (~50% type II fibers) (Henriksson‐Larsen et al. 1985) will have a stronger relationship with cBIS ECF followed by *T*_2_ of medial and/or lateral gastrocnemius (~43% type II fibers) (Henriksson‐Larsen et al. 1985) and the weakest association with the soleus muscle (20% type II fibers) (Gollnik et al. 1974).

As an alternative, we hypothesized that the muscle occupying maximum volume would also encompass the most interstitial fluid volume. Based on this assumption we hypothesized that the *T*_2_ of soleus muscle will have the highest correlation with cBIS ECF followed by medial and lateral gastrocnemius and TA, respectively (Albrachta et al. 2008). Henriksen et al. (1993) observed an increase in the rat soleus interstitial fluid volume following suspension or space flight. The authors attributed this increase in the interstitial fluid volume to atrophy of the muscles due to suspension. In contrast, the same study observed an increase in the extensor digitorum longus muscle wet weight attributed to hypertrophy of this muscle. Hence our a priori hypothesis lends greater support to the volume theory. For the purposes of this study *T*_2_ was considered as the reference method (gold standard) for comparison with cBIS ECF: ICF ratio in healthy and cBIS ECF in participants on HD.

For the healthy population we explored the association between *T*_2_ ECF: ICF ratio (Hatakenaka et al. 2006) to establish direct proportionality with the cBIS ECF. For participants on HD, as the treatment targets change in ECF only, we chose to explore the association between *T*_2_ and cBIS ECF before and after HD assuming no change in ICF.

To further investigate the association between the cBIS and MRI‐acquired measures, we compared the equality of the pre‐ and post‐HD coefficients of regression quantifying the mean rate of change in *T*_2_ of the muscles for each unit of cBIS ECF.

## Methods

### Participant eligibility

Participants' eligibility criteria were the age of 18 years; understood English and were able to provide informed consent; had no documented evidence of any disease impacting the nervous system and had no any condition that would preclude them from having an MRI or strength measurements, For the experimental group, the participants had to be stable on HD treatment for at least 3 months.

Participants not meeting all of the above mentioned inclusion criteria were excluded from the study. Healthy participants were recruited from the community and participants on HD were recruited from dialysis units affiliated with London Health Sciences Center. Western University's Ethics Review Board approved the study and all participants provided written informed consent prior to participation.

### Skeletal muscles

TA, LG, MG, and soleus muscles were investigated. These muscles represented anterior and posterior compartment of the calf (Gray 1918).

### Study protocol

Participants were positioned supine on a bed for 30 min to allow redistribution of water in the lower extremities prior to MRI (Berg et al. 1993). Measurements of hydration using BIS were collected while the participants lay supine in preparation for MRI. Data for healthy participants were acquired on a single occasion and for the participants on HD it was collected before and after a HD treatment session. The post‐HD data were collected after a minimum of 4 h following the treatment.

### Outcome measures

*Calf Extracellular Fluid:* A multi‐frequency BIS device (XiTRON 4200, Xitron Technologies, San Diego, CA) was used for automatic sequential measurements of calf segment with frequencies ranging from 5 kHz to 1 MHz. Two measuring (E_S1_ and E_S2_) and two injecting electrodes (E_I1_ and E_I2_) were placed on the lateral side of the tested leg. First E_SI_ electrode was placed at maximum circumference of the calf; E_S2_ was placed 10 cm distal to E_S1_. Injecting electrodes E_I1_ was placed 5 cms proximal to E_S1_ and E_I2_ was placed 5 cm distal to Es_2_. A fiduciary marker (vitamin E capsule) was placed at the E_S1_ electrode site for identification of this first measuring electrode on MRI. Each measurement was repeated at least 10 times and the average value was used in subsequent computation of cBIS ECF. Calculations and curve fitting (Cole‐Cole model) for data collected were done offline as described by Zhu et al. (2004).

*Transverse Relaxation Times:* Magnetic resonances imaging‐acquired data were collected on a 3.0 Tesla Tim Trio whole‐body imaging system (Siemens, Erlangen, Germany) using an 8‐channel knee coil. A multi‐echo spin‐echo (8 echoes) volume {11 contiguous 3 mm transverse slices; 160 mm field of view; 384 × 384 matrix; TE (13.1 ms to 93.6 ms); TR = 1500 ms} were used to measure the *T*_2_ of the TA, medial and lateral gastrocnemius and soleus muscles. *T*_2_ was calculated with the OsiriX plugin “T2 Fit Map”.

**Figure 1.**
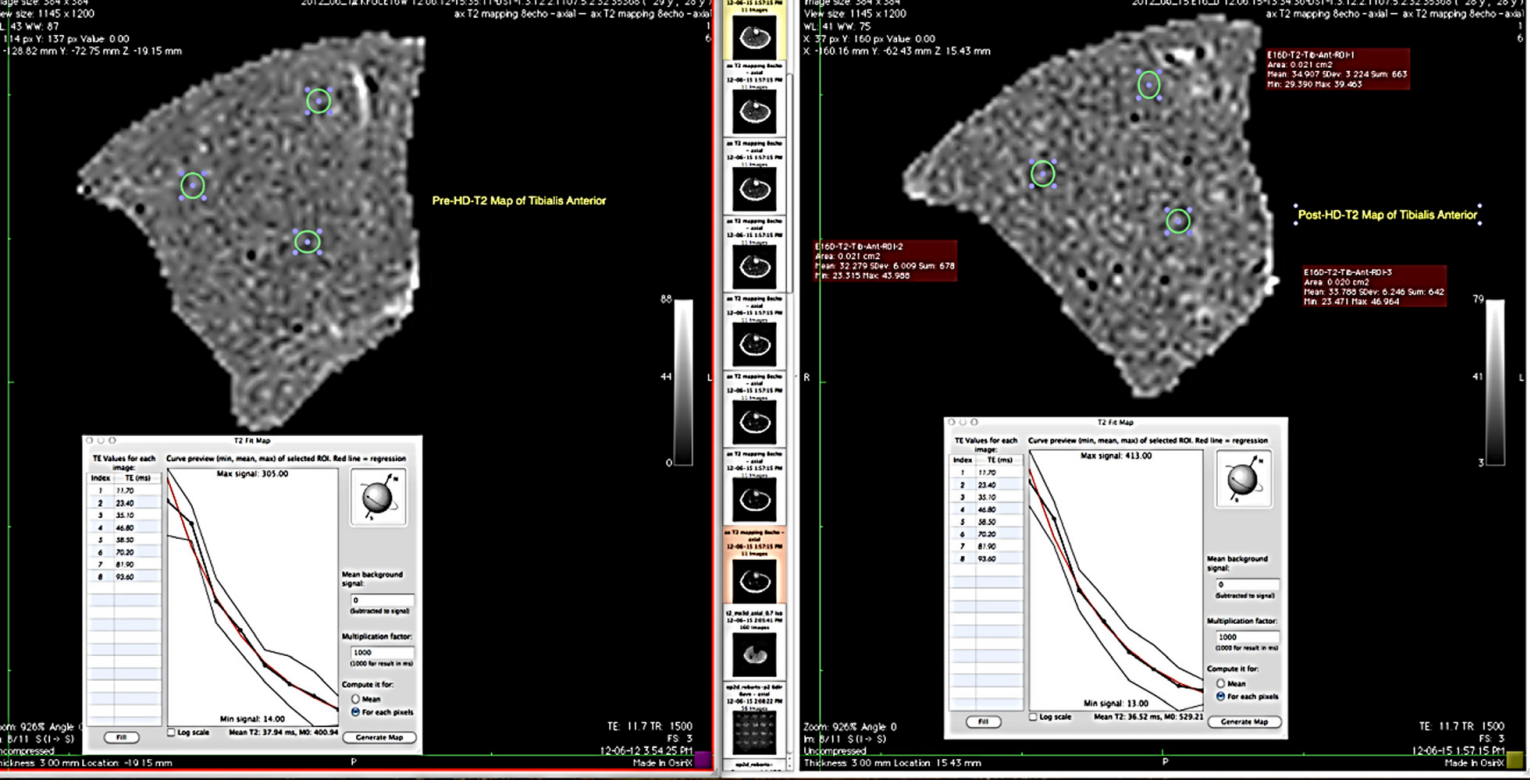
Example of measuring transverse relaxation times of Tibialis Anterior.

The muscles of interest were outlined close to the fiduciary marker visible on MRI, on the *T*_2_‐weighted images acquired for calculating the T2. The *T*_2_ maps for the muscle cross‐sectional area were generated in OsiriX using the “*T*_2_ Fit Map” plugin. On this map three areas or regions of interest (ROIs) less than 0.22 cm^2^ were selected taking care to avoid visible subcutaneous fats, septum, or neurovascular bundles (Fig. 1). Averages of three small areas of the muscle were calculated to obtain appropriate representation of the *T*_2_ for that muscle. Others have used a similar method of averaging *T*_2_ of two or three small ROIs for the determination of *T*_2_ of a muscle (Le Remeur et al. 1994).

### Related data

Physical activity of participants was quantified by using the Human Activity Profile Questionnaire (Fix and Daughton 1988) to identify differences in physical function between participants. The HAP consists of 94 activities, ranked in ascending order of difficulty according to the energy requirements (METs) of the task. The HAP has been validated against accelerometer values (an objective measure of physical activity) over a 7‐day period in people with ESRD/HD (r = 0.78) (Johansen et al. 2001). The presence of any comorbidity among the participants was quantified using the Charlson Comorbidity Index (Hall et al. 2004) calculated from the medical chart review of each participant.

### Statistical analysis

Descriptive data for the participants such as age, average body mass, body mass index, Human Activity Profile (Fix and Daughton 1988), and Charlson Comorbidity (Hall et al. 2004) scores were calculated as means and standard deviations. The associations between the outcome measures of hydration were evaluated using simple linear regression analysis. The equality of the before and after HD regression coefficients was assessed as described by Paternoster et al. (1998) for *N* = 4 degrees of freedom. The significance for the difference in equality of regression coefficients was determined only if the association between cBIS ECF and MRI measure was statistically significant before or after HD. A statistical software package (IBM SPSS v20.0) was used for all data analyses and Prism 4 for Macintosh (GraphPad Software Inc., San Diego, CA) was used for plotting the associations between the cBIS and MRI‐acquired measures. A *P*‐value of <0.05 was required for statistical significance.

## Results

We recruited 32 (16 men and 16 women) and 22 participants (11 men and 11 women) in the control and experimental groups, respectively. Table 1 summarizes the characteristics of the participants included in this study. Measures of hydration (cBIS ECF, ECF: ICF, and *T*_2_) collected in healthy and before and after HD are presented as means, and standard deviations in Table 2.

**Table 1.** Characteristics of participants included (*n* = 22).

Subject characteristics	Healthy participants Mean (SD)	Hemodialysis group Mean (SD)
Age (years)	50.8 (14.0)	50.6 (15.7)
Weight (kg)	75.27 (13.24)	75.1 (26.3)
Height (cm)	168.1 (8.6)	167.3 (12.7)
BMI (kg/m^2^)	26.3 (3.3)	26.68 (7.7)
Charlson Comorbidity Index	0.16 (0.72)	4.2 (2.4)
HAP‐MAS	87.8 (5.3)	69.3 (16.5)
HAP‐AAS	85.4 (8.3)	57.8 (16.7)
Number of medications	1 (1.33)	11.4 (2.4)
Cause of ESRD		Glomerulonephritis – 6 Polycystic Kidney Disease‐5 Hypertensive nephropathy‐4 Other nephropathies‐3 IgA nephropathy‐1 Vasculitis‐1 Rhabdomyolysis‐1 ESRD‐NYD‐1

AAS, adjusted activity score; BMI, body mass index; ESRD, end‐stage renal disease; HAP, human activity profile; NYD, not yet diagnosed; SD, standard deviation.

**Table 2.** Measures of hydration in healthy and hemodialysis group (before and after hemodialysis).

Measure	Healthy Mean (SD)	Pre‐HD Mean (SD)	Post‐HD Mean (SD)
BIS:ECF:ICF	0.31		
ECF (liters)		0.15 (0.05)	0.14 (0.05)
*T*_2_ (ms)			
TA	36.11	45.66 (6.83)	43.29 (6.17)
MG	38.94	43.81 (6.67)	43.29 (6.17)
LG	40.33	47.69 (6.89)	45.40 (6.19)
Soleus	39.82	45.47 (4.49)	42.82 (3.65)

BIS, bioelectrical impedance spectroscopy; ECF, extracellular fluid; HD, hemodialysis; LG, lateral gastrocnemius; MG, medial gastrocnemius; mm, millimeters; ms, milliseconds; TA, tibialis anterior; SD, standard deviation; *T*_2_, transverse relaxation time constant.

The pair‐wise comparisons for pre‐ and post‐HD measures of cBIS ECF were not significant with a mean difference of 0.02 L. However, the mean difference, pre‐ and post‐HD, for the *T*_2_ of TA, LG, MG, and soleus ranged from 2.5 to 5.2 ms; *P* < 0.05.

We explored the agreement between *T*_2_ of all calf muscles investigated and cBIS measures in healthy and HD population on both occasions; before and after HD. The *T*_2_ of TA, LG, MG, and soleus had a moderate correlation with cBIS ECF: ICF ratio in the healthy population (*β *= 0.622, *P* = 0.008) confirming a direct association with ECF of a muscle. The overall model in the healthy population explained for ~ 39% of variance in the cBIS‐acquired ECF: ICF ratio of the calf segment [*R*^2^ = 0.39, *F*_(4,27)_ = 4.287, *P* = 0.008]. For the HD group, the association between *T*_2_ of TA, LG, MG, and soleus and cBIS ECF were as follows: pre‐HD *β *= 0.039, *P* = 0.572; post‐HD *β *= 0.598, *P* = 0.094. The overall model explained variance of ~ 15% pre‐HD [*R*^2^ = 0.15, *F*_(4,17)_ = 0.749, *P* = 0.572], and ~35% post‐HD[*R*^2^ = 0.15, *F*_(4,17)_ = 0.749, *P* = 0.572], in the cBIS‐acquired ECF.

### Association between *T*_2_ of TA and calf BIS measures

In the healthy population the association between *T*_2_ of TA and cBIS ECF: ICF [*β *= 0.30, *P* = 0.09; b = −0.22, (95% CI: −0.85, 0.40), *t*_(30) _= −0.729, *P* > 0.05] was not significant and explained ~9% variance [*R*^2^ = 0.09, *F*_(1,30)_ = 3.009, *P* > 0.05] (Table 3). For the HD group, cBIS ECF was significantly associated with *T*_2_ of TA [*β *= 0.44, *P* = 0.042; b = 34.2 (95% CI: 26.0, 42.4), *t*_(20)_ = 8.7, *P* < 0.05] following HD only; cBIS ECF explained a significant proportion of variance in the *T*_2_ of TA [*R*^2^**=**0.19, *F*_(1,20)_ = 4.72, *P* = 0.042]. The association between before‐HD cBIS ECF and *T*_2_ of TA was nonsignificant (Table 4).

**Table 3.** Results of association between ratios of Calf BIS‐acquired ECF and ICF and MRI‐acquired measures of hydration in healthy population (*n* = 32).

	*R*	*R* ^2^	SEE	Constant
cBIS ECF/ICF				
TA – *T*_2_	0.30	0.09	0.07	−0.223 −0.85, 0.4
LG – *T*_2_	0.37*	0.14	0.07	−0.4 −0.37, 0.28
MG – *T*_2_	0.43*	0.19	0.07	−0.34 −0.3, 0.23
Sol – *T*_2_	0.60*	0.36	0.06	−0.26 −0.55, 0.21

cBIS, calf bioelectrical impedance spectroscopy; CI, confidence interval; ECF, extracellular fluid; HD, hemodialysis; LG, lateral gastrocnemius; MG, medial gastrocnemius; *T*_2_, transverse relaxation times; TA, tibialis anterior; Sol, soleus; SEE, standard error of estimate.

* *P* < 0.05.

**Table 4.** Results of association between Calf BIS ECF and MRI‐acquired measures of hydration in people on hemodialysis (*n* = 22).

	*R*		*R* ^2^		SEE		Constant	
	Pre‐HD	Post‐HD	Pre‐HD	Post‐HD	Pre‐HD	Post‐HD	Pre‐HD (95% CI)	Post‐HD (95% CI)
cBIS‐ECF								
TA – *T*_2_	0.29	0.44*	0.09	0.19	0.05	0.04	0.05 −0.1, 0.2	0.001 −0.13, 0.14
LG – *T*_2_	0.02	0.30	0.00	0.09	0.05	0.04	0.14 0.01, 0.29	0.037 −0.116, 0.19
MG – *T*_2_	0.18	0.47*	0.03	0.22	0.05	0.04	0.08 −0.78, 0.25	−0.01 −0.15, 0.88
Sol – *T*_2_	0.18	0.09	0.03	0.01	0.05	0.04	0.05 −0.2, 0.3	0.09 −0.18, 0.36

cBIS, calf bioelectrical impedance spectroscopy; CI, confidence interval; ECF, extracellular fluid; HD, hemodialysis; LG, lateral gastrocnemius; MG, medial gastrocnemius; *T*_2_, transverse relaxation times; TA, tibialis anterior; Sol, soleus; SEE, standard error of estimate.

* *P* < 0.05.

### Association between *T*_2_ of lateral and medial gastrocnemius, and soleus and calf BIS ECF

**Figure 2.**
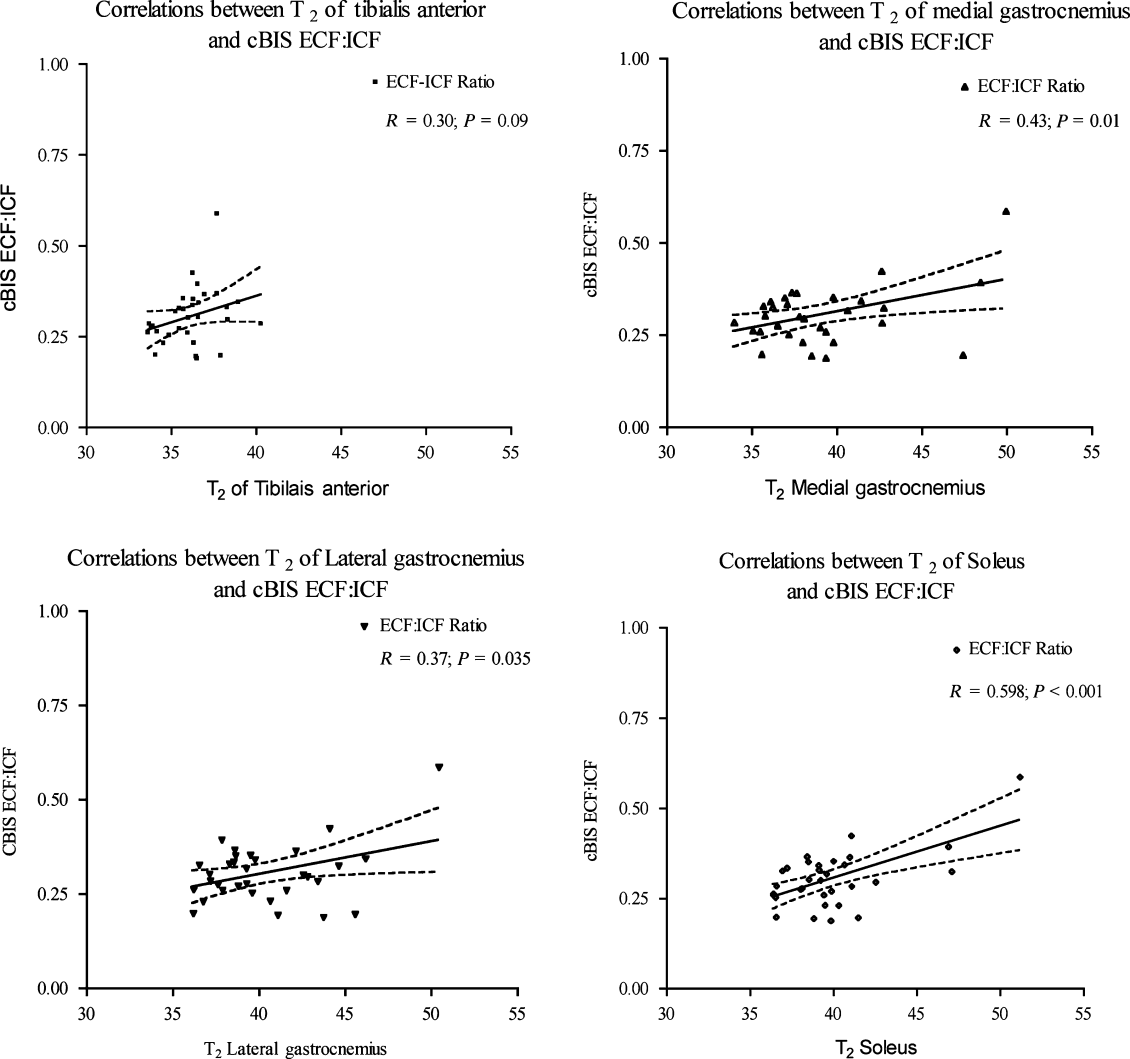
Pre‐ and post‐HD correlations between BIS and *T*_2_ of calf muscles in healthy populations.

In the healthy population the association between *T*_2_ of LG, MG, and soleus and cBIS ECF: ICF were as follows LG: *β *= 0.37, *P* = 0.035 [b = −0.44, (95% CI: −0.37, 0.28), *t*_(30) _= −0.277, *P* > 0.05]; MG: *β*=0.43, *P* = 0.014 [b = −0.03, (95% CI: −0.3, 0.23), *t*_(30) _= −0.258, *P* > 0.05]; Soleus: *β *= 0.598, *P* < 0.001 [b = −0.26, (95% CI: −0.55, 0.02), *t*_(30) _= −1.889, *P* > 0.05]. *T*_2_ of LG [*R*^2^ = 0.14, *F*_(1,30) _= 4.905, *P* = 0.035], MG [*R*^2^ = 0.19, *F*_(1,30) _= 6.866, *P* = 0.014] and soleus [*R*^2^ = 0.36, *F*_(1,30) _= 16.743, *P* < 0.001] explained a significant proportion of variance in the cBIS ECF: ICF ratio (Fig. 2).

**Figure 3.**
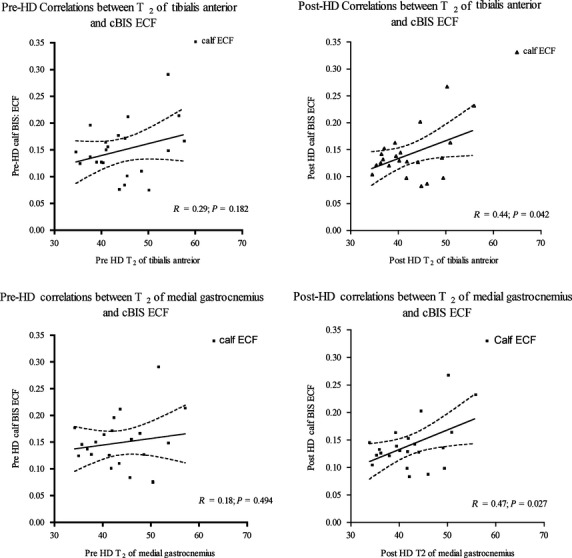
Pre‐ and post‐HD Associations between Calf ECF and *T*_2_ of Tibialis Anterior and Medial gastrocnemius. cBIS, calf bioelectrical impedance spectroscopy; ECF, extracellular fluid; Med, medial; Gas, gastrocnemius; *T*_2_, transverse relaxation times.

For the ESRD group, cBIS ECF was significantly associated with *T*_2_ of MG [*β *= 0.47, *P* = 0.027; b = 33.6 (95%CI: 25.5, 41.6), *t*_(20)_ = 8.68, *P* < 0.05]; following HD only; *T*_2_ of MG explained a significant proportion of variance in the cBIS ECF [*R*^2^ = 0.22, *F*_(1,20) _= 5.69, *P* = 0.027] (Fig. 3). The associations between the cBIS ECF and *T*_2_ were nonsignificant before HD. Following HD, the associations between *T*_2_ of lateral gastrocnemius and soleus and cBIS ECF were nonsignificant as well.

### Effect of HD on associations between MRI‐acquired *T*_2_ and Calf BIS ECF

The before and after HD coefficients of regression determining prediction of *T*_2_ of TA (*t*_(18) _= 2.175; *P* < 0.05), medial gastrocnemius (*t*_(18) _= 4.785; *P* < 0.05) by cBIS ECF were significantly different.

## Discussion

The main purpose of this study was to examine the association between the MRI‐acquired *T*_2_ and calf BIS methods for estimating hydration for the TA muscle in healthy and people with ESRD/HD. The results of the study indicated that the association between *T*_2_ of TA in healthy population was not significant. The association between *T*_2_ of TA and cBIS ECF were significant following HD only. For our secondary objectives, the associations between the *T*_2_ of LG, MG, and soleus and calf BIS in the healthy population were small to moderate (ranging from 0.37 to 0.6); the *T*_2_ of the soleus muscle had the largest association with the cBIS measure. However, in people on HD the associations of *T*_2_ of medial gastrocnemius with cBIS ECF was significant following post‐HD only. The pre‐ and post‐HD coefficients of regression were significantly different for the muscles evaluated. These results suggest that cBIS‐acquired measures could be used to estimate hydration of LG, MG, and soleus in healthy population but cannot be used to estimate the change in hydration following HD treatment in people with ESRD. These findings of small to moderate correlations between *T*_2_ of LG, MG, and soleus and cBIS ECF: ICF ratio and nonsignificant correlation of cBIS ECF: ICF with *T*_2_ of TA in healthy population support our a priori hypothesis based on muscle volume theory, that is, the larger the muscle volume the greater ECF it will encompass; in our study the largest muscle was soleus with largest correlation of 0.6.

To our knowledge, this is the first study that has explored the association between *T*_2_ and cBIS ECF of the calf muscles. For the healthy populations our results show a comparable correlation as reported in the literature by Hatakenaka et al. (2006); whereas the correlation between *T*_2_ and cBIS ECF volume in people with ESRD/HD were smaller. This could be due to several factors including the interval between the healthy and pathological state of the skeletal muscle (administration of steroids to induce myopathy for the study by Hatakenaka et al. (2006) and duration on HD treatment in our participants). Hatakenaka et al. (2006) evaluated the *T*_2_ relaxation times following 6 weeks of steroid administration. This can be described as early phase of fast progressive atrophy (Adami et al. 2007). All of our participants had been on HD for a period greater than 3 months; a chronic phase where atrophic muscle fibers have been replaced with fibrous or lipid tissue (Adami et al. 2007). Hence the contribution of the lipids replacing the atrophic muscles in our ESRD/HD participants to the *T*_2_ of the muscles may have influenced its association with cBIS ECF; we controlled for this by choosing to select small regions of interest (ROIs) to estimate the muscle *T*_2_ time constants

Our findings of a nonsignificant dorrelation between *T*_2_ and calf ECF:ICF in healthy popultion lend support to our volume theory; TA has the smallest muscle volume and hence the smallest correlation of 0.2. However, in the people with ESRD, *T*_2_ of TA explained ~ 20% (*R*^2^ = 0.19) of the observed variation in cBIS ECF following HD. Generally *T*_2_ values correlate most strongly with bulk water content of the tissue (Baulby and Rugg‐Gunn 2004). Additionally, tissues with high concentrations of lipid protons have longer *T*_2_. Such factors related to skeletal muscle composition and structure that impact *T*_2_ may explain our results of ~ 20% variation in cBIS ECF by *T*_2_ of TA in people with ESRD/HD.

Using customized software for MRI images of the ankle muscles to differentiate between contractile and noncontractile areas of ankle muscles in participants on HD, Johansen et al. (2003) were able to show an increase in total noncontractile area. This noncontractile area could consist of lipid deposition and the impact the *T*_2_ values of TA and consequently associations between *T*_2_ of TA and cBIS ECF (pre‐HD there was a larger ECF in relation to muscle (solids) proteins and post‐HD relatively less ECF). This post‐HD change in ECF of TA in relation to the muscle solids (proteins) may have impacted the association between the *T*_2_ of TA and cBIS ECF.

Our finding supporting the volume theory (largest association with soleus in healthy population) is contrary to the findings of largest association with *T*_2_ of TA and MG following HD in people with ESRD/HD (muscles with 47–50% Type II fibers). We did not find any studies reporting the morphological characteristics of the soleus muscle in people with ESRD/HD. Interestingly two studies evaluating the morphological characteristics of MG using needle biopsy in this population observed either predominance or hypertrophy of Type I fibers (Sawant et al. 2011). Whether factors related to vascular perfusion, electrolyte imbalance, or atrophy of skeletal muscle in people with ESRD/HD impacted the *T*_2_ a values/cBIS measure requires to be investigated further.

Factors related to cBIS ECF as well may contribute to the variations in its associations with *T*_2_ of TA in the healthy and experimental populations. For the cBIS ECF, this includes plasma in the large vessels viz. the popliteal artery, anterior tibial artery, and saphenous veins, interstitial spaces embedded within the other muscles of the calf viz. LG, MG, soleus, and peronei. The variation in the cBIS ECF associated with TA may perhaps also be related to the total space occupied by the muscle in relation to the total calf volume and variations in *T*_2_ of lateral and medial gastrocnemius and soleus may contribute to variations in cBIS ECF as observed in our exploratory model evaluating the association between *T*_2_ of all muscles and cBIS ECF.

Kaysen et al. (2005) used whole‐body BIS to show that ICF volume does not vary significantly during interdialytic periods. According to Charra (2007) this expansion of ECF appears to be largely corrected for the excess plasma volume following the HD sessions. During the few hours of HD treatment the plasma compartment is ultrafiltered down to its normalized volume. These reports suggest that the excess plasma volume in relations to the muscle proteins (solids) largely confounded the associations between interstitial fluid measured using *T*_2_ and cBIS ECF prior to HD treatment. Measurement bias at higher values of ratio of ECF and ICF may also have contributed to poor associations prior to HD treatment (Sawant et al. 2013). This supports our findings of nonsignificant associations between the measures of individual skeletal muscle hydration obtained using MRI and BIS, before HD in participants with ESRD. Skeletal muscle is a heterogeneous structure in its composition and architecture. Measurement of hydration of a limb segment such as calf using BIS is considered to be at a “whole body” level, whereas MRI estimates hydration at the “tissue system level” (Heymsfield et al. 1995). A direct comparison between these two techniques, based on different assumptions and methods is challenging. No study has yet determined a direct relationship between *T*_2_ and wet/dry weight of the calf/shank muscles in people with ESRD/HD.

Our findings of prolonged *T*_2_ values for all the muscle before and after HD in people with ESRD/HD confirm prior observations of chronic expansion of ECF in this population. Besides atrophy of the muscles may leads to reductions in proteins/solids in comparison to the water or lipid contents and impact the *T*_2_ values. The post‐HD association of *T*_2_ of all the muscles evaluated in this study viz. TA. MG. LG and soleus to cBIS ECF (*β *= 0.598, *P* = 0.094) was comparable to the association between *T*_2_ of all muscles and ratio of cBIS ECF in healthy population (*β *= 0.622, *P* = 0.008). This is in accordance to the earlier reports that suggest correction of ECF in HD population comparable to the healthy population.

The HAP‐AAS (the best estimate of respondents’ average level of energy expenditure in comparison with peers of same age) of the control participants in this study was within the range reported for healthy population. For the HD group, the HAP‐AAS scores indicate that the participants included in this study were functioning at ~61% of the maximum possible activity level or at below‐average fitness level (Sharrock et al. 1972; Fix and Daughton 1988). The participants on HD in this study had a comorbidity score greater than three (the mortality rate was zero for ESRD/HD cohort with a Charlson Comorbidity Index score of three) (Di Iorio and Cillo 2004). Since the comorbidity scores correlate with the phase angle (suggesting an altered intra and extra cellular water distribution) of BIS and functional levels (Norman et al. 2010), these results can be applied to participants with HAP‐AAS and comorbidity scores similar to participants in this study.

## Conclusions

In conclusion, this is the first study that has looked at the associations between the hydration measures of the calf muscles acquired using MRI and BIS. Although cBIS ECF can be used for estimation of ECF in the calf segment, *T*_2_ of a calf muscle provides estimates of individual skeletal muscle hydration before and after HD treatment. Calf BIS could be used to estimate the skeletal muscle hydration of LG, MG, and soleus muscles in healthy population. However, Calf BIS cannot be used to estimate hydration of the single calf muscle in people with ESRD/HD.

Our findings of the overall model exploring correlations between cBIS ECF and *T*_2_ of all the calf muscles evaluated require to be interpreted with caution due to relatively low numbers of test subjects and hence limited statistical power. The characteristics of the participants on HD included in this study were comparable to those reported in literature and hence the results of this study can be applied to a broader population on HD except for those with neuromuscular disorders.

## Acknowledgments

The Renal Research Institute of New York for providing training, software and bioelectrical impedance spectroscopy equipment to measure calf hydration. Dr. Andrew Johnson, Chair, Health and Rehabilitation Sciences, Western University, London, ON, for advice on statistical analysis. Kim Krueger and Oksana Opaleych for their patience while I worked on refining the protocol. All the participants for their time and support for this study.

## Conflict of interest

Anuradha Sawant was a recipient of the Allied Health Doctoral Fellowship from The Kidney Foundation of Canada. The authors declare no conflicts of interest.
